# MUC1 Immunohistochemical Expression as a Prognostic Factor in Gastric Cancer: Meta-Analysis

**DOI:** 10.1155/2016/9421571

**Published:** 2016-04-17

**Authors:** Xiao-Tong Wang, Fan-Biao Kong, Wei Mai, Lei Li, Li-Ming Pang

**Affiliations:** ^1^Departments of Gastrointestinal and Peripheral Vascular Surgery, People's Hospital of Guangxi Zhuang Autonomous Region, Nanning 530021, China; ^2^Department of Surgery, The First Affiliated Hospital of Guangxi University of Chinese Medicine, Nanning 530023, China

## Abstract

MUC1, a member of the mucin family, is expressed in tumors of various human organs and may function as an antiadhesion molecule that inhibits cell-to-cell adhesion, inducing tumor metastasis, and served as a potential biomarker of tumor progression in early gastric cancer. However, its prognostic significance in gastric cancer is still in dispute. We performed a meta-analysis to evaluate the relationship between MUC1 expression and prognosis of gastric cancer. A total of ten eligible studies with 834 cases and 548 controls were included. MUC1 positive cases were highly positive in intestinal-type carcinomas (OR = 1.76, 95% CI: 1.27–2.44, *P* = 0.0008 fixed-effect), higher rate of vascular invasion (OR = 1.64, 95% CI: 1.13–2.39, *P* = 0.009 fixed-effect), and lymph node metastasis (OR = 2.10, 95% CI: 1.20–3.67, *P* = 0.01 random-effect), as well as lower 5-year survival rate (HR = 0.27, 95% CI: 0.11–0.66, *P* = 0.004 random-effect). However, the presence of MUC1 was not associated with gender, tumor size, histologic differentiation, and clinical stage. In summary, MUC1 is a prognostic factor in gastric cancer, which acts as a marker of poor outcome in patients with gastric cancer. Further clinical studies are needed to confirm the role of MUC1 in clinical practice.

## 1. Introduction

Despite overall decline in gastric carcinoma (GC) prevalence, it is still the second most frequently malignant tumor in Eastern Asia, particularly in China [1, 2]. Patients with gastric cancer have excellent survival if there is no regional lymph node involvement [3]. Unfortunately, gastric cancer is difficult to be diagnosed at an early stage. As a result, there is great interest in finding a prognostic marker for this potentially curable group of patients.

Mucins are a group of high molecular weight glycoproteins, and nine apomucins (MUC1-8 and MUC5B) have been identified to date. MUC1 is a membrane-bound mucin, which plays an important role in the impairment of cell-cell adhesion, the immune response, and/or altered intracellular signaling, and is involved in the development and progression of gastric cancer [4]. A number of reports have demonstrated that MUCl antigen is expressed in human gastric cancer, and it has been shown to be an indicator of clinicopathological significance of gastric carcinomas [5–8]. However, the relation between MUC1 expression and clinicopathological features remains controversial. So far several studies have demonstrated that MUC1 positive expression in gastric cancer was determined to be statistically significant, with worse differentiation and higher rate of lymph node metastasis [9–11]. However, Kocer and colleagues showed that there was no association between MUC1 expression and lymph node metastasis of gastric carcinoma [12]. Therefore, because of the limits of the single study and insufficient samples, we conducted a meta-analysis to investigate the correlation between MUC1 and prognosis of GC and to consider MUC1 expression as a novel prognostic biomarker for survival in GC patients.

## 2. Methods

### 2.1. Literature Search Strategy

PRISMA statement is performed in this meta-analysis [19]. We carefully searched the relevant studies (published before 10 November 2015) that investigated the prognostic value of MUC1 expression in gastric cancer from Web of Science, Embase, PubMed, MEDLINE, and Cochrane Library. The language of articles was limited to English. Studies were selected using following key terms: (MUC OR MUC-1 OR MUC1) AND (Gastric carcinoma OR GC OR Gastric cancer) AND (prognosis OR prognostic OR outcome OR mortality OR survival). The references of manuscript were also examined to confirm potential studies. XTW and FBK conducted the search and assessed the eligibility of studies independently.

### 2.2. Study Selection

Inclusion criteria were as follows. (1) The study must explore the association between MUC1 and human gastric cancer. (2) The species must be human. (3) Tumors were pathologically verified. (4) Articles provided sufficient data to evaluate odds ratio (OR), hazard ratio (HR), and their 95% confidence interval (CI). (5) Articles focused on relationship between MUC1 and GC clinicopathological character. (6) MUC1 expression was assessed by immunohistochemistry (IHC). (7) MUC1 expression was estimated in GC samples. (8) Not any form of preoperative neoadjuvant therapy was received.

Exclusion criteria were as follows: (1) publication that was of nonresearch articles; (2) data duplication or missing important information for this meta-analysis; and (3) studies that were based on animal or human cell lines. If we needed additional information and data which cannot be found in article, we will email the authors for further information.

### 2.3. Data Acquisition and Quality Assessment

Relevant characteristics and outcome data were collected by two independent reviewers. The main characteristics of articles were listed as follows: (1) first author; (2) year of publication; (3) country; and (4) median follow-up. The relevant clinical data of studies included (1) patients' number; (2) gender (male/female); (3) age (years); (4) pathological pattern; (5) histologic origin; (6) antibody source; (7) dilution; (8) evaluation method of MUC1 expression level; and (9) low versus high MUC1. HR of 5-year survival rate was first calculated from multivariable analysis or Kaplan-Meier survival curves in article. Any disagreement was resolved by discussion. The quality of each of the available studies in our analysis was assessed by Newcastle-Ottawa Scale (NOS).

### 2.4. Statistical Analysis

OR with 95% CI was performed to combine the pooled data. Heterogeneity was tested by the *Q* test which was considered statistically significant when *P* values < 0.01 and inconsistency index *I* ^2^ statistic which takes values between 0% and 100% (*I* ^2^ < 25%, low heterogeneity; *I* ^2^ = 25%–50%, medium heterogeneity; *I* ^2^ = 50%–75%, high heterogeneity; *I* ^2^ = 75%–100%, resp., heterogeneity). According to the heterogeneity of studies, it is considered to be significant when *P* < 0.01 or *I* ^2^ > 50%; the random-effects model (based on DerSimonian and Laird method) or fixed-effects model (based on Mantel-Haenszel method) was used for meta-analysis [20]. The data on the predictive ability of MUC1 overexpression for 5-year survival rate were combined across studies using fixed- and random-effect models for the synthesis of hazard ratio (HR). The HR of 5-year survival rate was calculated from the reported data directly by number of events within 5 years after surgery was used or data reading from Kaplan-Meier survival curve. The funnel plot was examined to explore the possibility of publication bias [21].

Kaplan-Meier curves were read by Engauge Digitizer version 2.11. *P* < 0.05 was considered as statistically significant publication bias. All of the calculations were performed by Review Manager 5.2 (RevMan version 5.2 (Copenhagen); the Nordic Cochrane Centre, the Cochrane Collaboration).

## 3. Results

### 3.1. Eligible Studies

**Figure 1:**
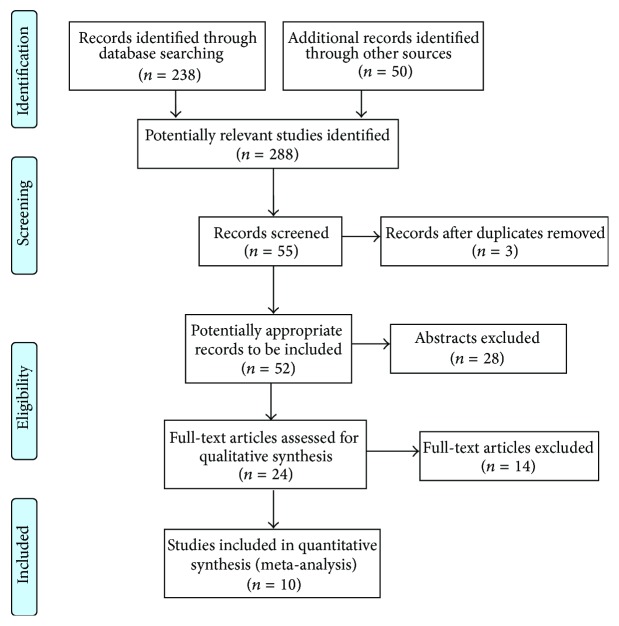
Flowchart of the search process.

As shown in Figure 1, our initial search yielded 238 studies. According to the inclusion and exclusion criteria, 10 papers [9–18] were recruited into our meta-analysis. Studies were carried out in China, Turkey, Korea, Japan, and Taiwan.  Table 1 presents the study characteristics for the included trials.

### 3.2. High MUC1 Expression and Prognosis of GC

**Figure 2:**
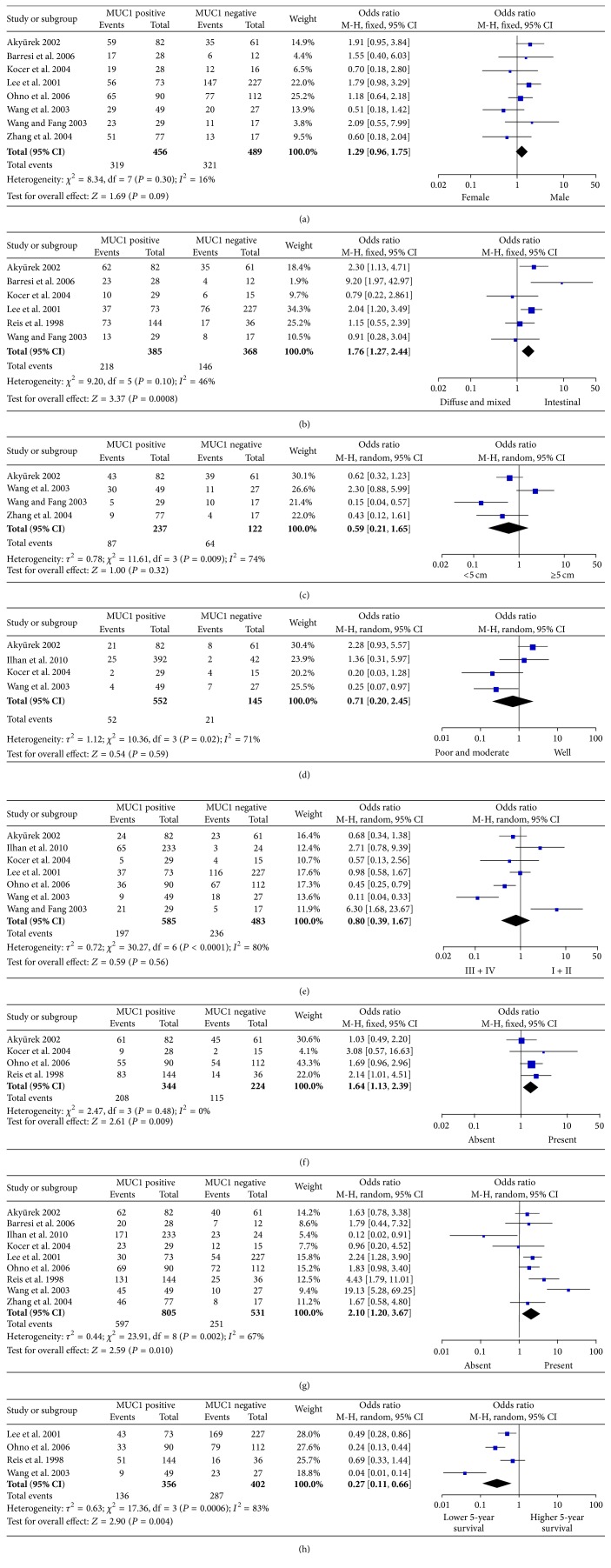
Forest plot of OR was assessed for association between MUC1 and clinical pathologic features, such as gender (a), Lauren classification (b), tumor size (c), tumor differentiation (d), clinical stage (e), vascular invasion (f), lymph node metastasis (g), and 5-year survival rate (h).

The putative MUC1 was not associated with gender (pooled OR = 0.59, 95% CI: 0.96–1.75, *P* = 0.09 fixed-effect), tumor size (pooled OR = 1.29, 95% CI: 0.21–1.65, *P* = 0.32 random-effect), tumor differentiation (OR = 1.58, 95% CI: 1.13–2.21, *P* = 0.007 fixed-effect), and clinical stage (OR = 0.80, 95% CI: 0.39–1.67, *P* = 0.59 random-effect) (Figures 2(a), 2(c), 2(d), and 2(e)). However, MUC1 expression in gastric cancer was associated with biologically aggressive phenotypes such as vascular invasion (OR = 1.64, 95% CI: 1.13–2.39, *P* = 0.009 fixed-effect) and lymph node metastasis (OR = 2.10, 95% CI: 1.20–3.67, *P* = 0.01 random-effect) (Figures 2(f) and 2(g)). In accordance with the Lauren classification, the expression rate of MUC1 in intestinal-type carcinomas was significantly higher than that in diffuse-type carcinomas (pooled OR = 1.76, 95% CI: 1.27–2.44, *P* = 0.0008 fixed-effect) (Figure 2(b)). Analysis of these data showed that MUC1 was highly correlated with lower 5-year survival rate (pooled OR = 0.27, 95% CI: 0.11–0.66, *P* = 0.004 random-effect) (Figure 2(h)).

### 3.3. Publication Bias

**Figure 3:**
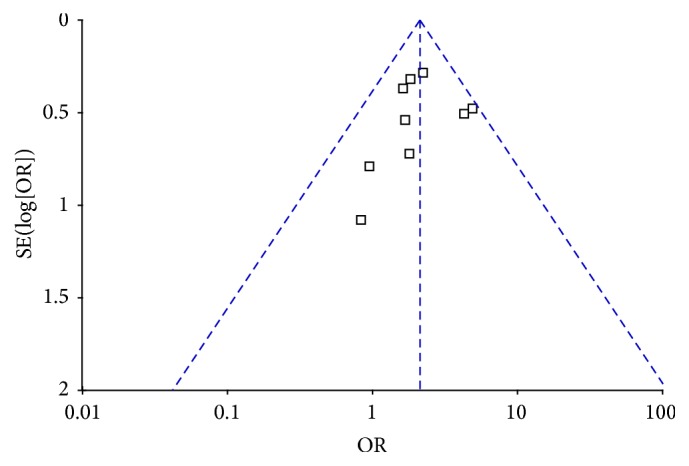
Funnel plot of studies to detect publication bias.

The interpretability of publication bias was assessed by using the inverted funnel plot approach recommended for meta-analyses [22]. As shown in Figure 3, no publication bias was detected in all comparisons.

## 4. Discussion

Although the risk of GC has dropped off in recent years, it remains the fifth most common malignant neoplasm around the world [23]. Some clinical studies revealed that the prognosis of GC patients depends on the tumor histological morphology and tumor-node-metastasis (TNM) classification [24–26]. However, there are not any GC-specific markers to predict the gastric cancer metastasis and survival [27]. Therefore, it is essential to detect an established biomarker to investigate the progression of gastric carcinomas and improve treatment of GC patients.

There is some indication that MUC1 expression is involved in tumor progression as well. MUC1, an epithelial mucin glycoprotein, is highly expressed in lactating mammary glands [28]. Some researches have shown that alterations of mucin expression take place in gastric carcinomas [29]. Under pathological conditions, such as colon adenocarcinoma and pancreas adenocarcinoma or stomach adenocarcinoma, MUC1 would change its expression fashion and rate [8]. In previous reports, gastric carcinomas were found to contain a higher level of MUC1 mucin expression than normal gastric mucosa [18, 30, 31]. Lee et al. [11] found that MUC1 positive Korean patients suffering from gastric carcinoma showed significantly poorer survival than those negative for MUC1. Utsunomiya et al. and Baldus and colleagues also found that MUC1 expression was associated with a poor outcome, irrespective of its glycosylation status, as assessed by the use of monoclonal antibodies recognizing only core peptides [32, 33]. They indicated that MUC1 was an independent prognostic indicator for gastric carcinoma. It can be concluded that MUC1 expression was a precursor of gastric carcinoma and served as a reliable tumor marker in gastric cancer.

However, other studies showed that no significant correlation could be determined between MUC1 and clinicopathological parameters [12, 17]. These researches suggested that MUC1 did not affect the progression of human gastric cancer. These conflicting results were likely due to small sample size of the study.

Meta-analysis was originally developed to combine the results of randomized controlled trails, and recently this approach has been applied successfully for identification of prognostic indicators in patients with malignant diseases [34, 35]. This meta-analysis is the first study to systematically estimate MUC1 expression and its relationship with the patients' clinicopathological characteristics. Statistical significance was reached that MUC1 overexpression, as detected by immunohistochemistry, was significantly associated with vascular invasion, lymph node metastasis, and 5-year survival. We observed a correlation of MUC1 positivity with higher rate of vascular invasion and lymph node metastasis. These findings indicated that MUC1 positive might be related to invasiveness of gastric carcinoma cells, which is in accordance with the experimental results that MUC1 positive tumors were associated with synchronous liver metastasis and overexpression of MUC1 in carcinoma cells decreases cell-cell interaction and increases the metastatic capacity of carcinoma cells, favoring invasion of tumor cells into the underlying stroma, lymph, and blood vessels [36]. Moreover, although there was not a significant correlation of MUC1 positive with tumor size, tumor differentiation, and clinical stage, it still had the tendency toward higher expression in advanced stage of cancer. The reason for this result may be too small sample size included in the meta-analysis. In addition, MUC1 positive gastric cancer patients also displayed lower 5-year survival rate than MUC1 negative ones. Therefore, high levels of MUC1 expression were found to be related to poor prognosis, as was the case in the present study.

There were several limitations in our meta-analysis. First of all, only published studies were included in the meta-analysis. Therefore, publication bias may have occurred, even though the use of a statistical test did not show it [37]. We tried to retrieve all relevant data that was not available from the published reports, but it is unavoidable that some data could still be missing. Missing information may reflect “negative” or more conservative association of MUC1 with clinicopathological parameters that could reduce the significance of MUC1 expression as a predictor of outcome in gastric cancer. Second, non-English literature was included in our meta-analysis which leads to losing some potential important survival data; meanwhile there might be selection bias. Moreover, heterogeneity between studies was low for most of the dichotomous variables examined in this analysis but was marked for all the continuous variables. There was significant variability in terms of definitions, inclusion criteria, exclusion criteria, operating technique, and measurement of outcomes. It was not possible to match all patient groups for age, BMI, preoperative therapy, and previous abdominal history. All these factors may have contributed to the high heterogeneity between studies. Although RE model was used to decrease heterogeneity between studies, it is not totally ruled out. Finally, the available data do not evaluate whether MUC1 may influence the response to specific therapeutic regimens. Therefore, we minimized the bias by confirming a detailed protocol before initiating the study, by performing a careful search for published studies, and by using explicit methods for study selection, data extraction, and data analysis.

## 5. Conclusion

In conclusion, our meta-analysis suggests that MUC1 expression might be a marker of poor prognosis for survival in patients with gastric cancer, if detected by immunochemistry. However, because of the heterogeneities of included studies and bias of meta-analysis, our conclusions need to be interpreted with caution.

## Figures and Tables

**Table 1:** Study characteristics for the included studies.

Author (year, country)	Samples	Median age (range)	M/F	Adequacy of evaluating MUC1 expression	Study period	Median follow-up (months)	NOS score
Akyürek et al. [13] (2002, Germany)	143	NS	94/49	Yes	NS	30 (2–80)	9

Barresi et al. [14] (2006, Italy)	40	69.4 (54–77)	23/17	Yes	NS	NS	9

Ilhan et al. [9] (2010, Turkey)	257	NS	201/56	Yes	2000–2007	NS	8

Kocer et al. [12] (2004, Turkey)	44	59.7 (27–77)	31/13	Yes	1996–2001	25 (1–79)	7

Lee et al. [11] (2001, Korea)	59	NS	56/17	Yes	1995-1995	42 (1–60)	9

Ohno et al. [15] (2006, Japan)	202	63	142/60	Yes	1993–2000	NS	9

Reis et al. [16] (1998, UK)	180	NS	105/75	Yes	NS	NS	8

Wang et al. [17] (2003, China)	76	65 (32–84)	52/24	Yes	1996–1998	30 (1–58)	7

Wang and Fang [18] (2003, China)	46	54.6 (30–70)	34/12	Yes	NS	NS	8

Zhang et al. [10] (2004, China)	94	52.1 (25–75)	64/30	Yes	1989–2000	NS	8

NA: not available, M/F: male/female, and NOS: Newcastle-Ottawa Scale.
